# Association of fat-soluble vitamins (A, D, and E) status with humoral immune response to COVID-19 inactivated vaccination

**DOI:** 10.3389/fnut.2023.1167920

**Published:** 2023-05-16

**Authors:** Yao Deng, Liting Huang, Peixin Liu, Xuyang Geng, Zefang Lin, Zhixiong Zheng, Meixiao Zhan, Zhiren Zhang, Junwei Liu, Taoping Sun

**Affiliations:** ^1^Guangdong Provincial Key Laboratory of Tumor Interventional Diagnosis and Treatment, Zhuhai Precision Medical Center, Zhuhai People's Hospital, Zhuhai Hospital Affiliated with Jinan University, Zhuhai, China; ^2^Department of Orthopedic, Zhuhai People's Hospital, Zhuhai Hospital Affiliated with Jinan University, Zhuhai, China; ^3^Party Committee of the Communist Party of China, Zhuhai Health Bureau, Zhuhai, China

**Keywords:** SARS-CoV-2, Omicron, vaccine, neutralizing antibody, fat-soluble vitamins, cohort study

## Abstract

**Background:**

Fat-soluble vitamins (A, D, and E) are essential for the proper functioning of the immune system and are of central importance for infection risk in humans. Vitamins A, D, and E have been reported to be associated with the immune response following vaccination; however, their effects on the immune response to severe acute respiratory syndrome coronavirus 2 (SARS-CoV-2) vaccination remain unknown.

**Methods:**

We measured the neutralizing antibody titers against wild type and omicron within 98 days after the third homologous boosting shot of inactivated SARS-CoV-2 vaccine (BBIBP-CorV or CoronaVac) in 141 healthy adults in a prospective, open-label study. High-performance liquid chromatography-tandem mass spectroscopy was used to determine the concentrations of plasma vitamins A, D, and E.

**Results:**

We found that the anti-wide-type virus and anti-omicron variant antibody levels significantly increased compared with baseline antibody levels (P < 0.001) after the third vaccination. 25(OH)D_3_ was significantly negatively associated with the baseline anti-wide-type virus antibody concentrations [beta (95% CI) = −0.331 (−0.659 ~ −0.003)] after adjusting for covariates. A potentially similar association was also observed on day 98 after the third vaccination [beta (95% CI) = −0.317 (−0.641 ~ 0.007)]. After adjusting for covariates, we also found that 25(OH)D_3_ was significantly negatively associated with the seropositivity of the anti-omicron variant antibody at day 98 after the third vaccination [OR (95% CI) = 0.940 (0.883 ~ 0.996)]. The association between plasma 25(OH)D_3_ with anti-wild-type virus antibody levels and seropositivity of anti-omicron variant antibodies were persistent in subgroup analyses. We observed no association between retinol/α-tocopherol and anti-wide-type virus antibody levels or anti-omicron variant antibody seropositive in our study.

**Conclusion:**

The third inactivated SARS-CoV-2 vaccination significantly improved the ability of anti-SARS-CoV-2 infection in the human body. Higher vitamin D concentrations could significantly decrease the anti-wide-type virus-neutralizing antibody titers and anti-omicron variant antibody seropositive rate after the inactivated SARS-CoV-2 vaccination in people with adequate levels of vitamin D, better immune status, and stronger immune response; further studies comprising large cohorts of patients with different nutritional status are warranted to verify our results.

## 1. Introduction

Vaccination against the severe acute respiratory syndrome coronavirus 2 (SARS-CoV-2) is undoubtedly an effective means of mitigating the coronavirus disease 2019 (COVID-19) pandemic. Different people obtain varying levels of protection from vaccines; therefore, the identification of sensitivity factors that affect vaccine protection is essential. The current research has focused on the association of unhealthy lifestyles and disease status with the immunogenicity of SARS-CoV-2 vaccines (1, 2). However, studies on the association between nutritional status and the immunogenicity of SARS-CoV-2 vaccines are relatively scarce and have yielded inconsistent results.

The optimal status of specific micronutrients is crucial for maintaining immune components within normal activity and improving host defenses against infections. The fat-soluble vitamins A, D, and E are essential for the proper functioning of the immune system (3) and play key roles at every stage of the innate and adaptive immune responses. Recent studies suggest that vitamin A may positively or negatively affect vaccine antibody response. Previous animal experiments have revealed that an adequate level of vitamin A is necessary to mount an efficient antibody response to many antigens (4); however, it has also been found that vitamin A-deficient animals can produce a strong antibody response to some antigens (4, 5). Furthermore, some studies have reported that vitamin A supplementation can stimulate an antibody response to vaccination, even in animals with normal vitamin A levels (6, 7). A human clinical trial also showed that vitamin A supplementation could improve immune responses to influenza virus vaccines in vitamin A-insufficient children at the baseline (8).

The effect of vitamin D on the antibody response to different vaccines is inconsistent. Vitamin D enhances the vaccine antibody response to tetanus and hepatitis B (9, 10); however, it is negatively correlated with the antibody response to the human papillomavirus (HPV) vaccine (11). Some studies have suggested that vitamin D does not influence the antibody response to the influenza vaccine (12–14). Similarly, animal studies have observed that vitamin E supplementation increases the antibody response in poultry (15, 16). However, randomized clinical trials have revealed that vitamin E supplementation has no effect on the antibody response to tetanus toxoid and pneumococcal polysaccharide vaccines in humans (17, 18), and a population study also showed no association between serum vitamin E levels and influenza vaccine response (19). The nutritional status of vitamins has different effects on antibody responses to different antigens or vaccines. However, it is unknown whether vitamins A, D, and E affect the immune response to SARS-CoV-2 vaccines.

An ecological study demonstrated that the intake levels of relevant micronutrients, especially vitamin D, were inversely associated with COVID-19 incidence and mortality (20). Moreover, a nutrigenetic study has shown that micronutrients, including vitamins A and D, and relevant genetic factors can help strengthen the immune system of individuals and prepare populations to fight against COVID-19 (20). A recent study showed that serum-neutralizing antibody levels gradually decreased after the second dose of inactivated vaccines within half a year; therefore, a third booster dose is necessary to maintain the effectiveness of inactivated vaccines (21). With the repeated outbreaks of the COVID-19 epidemic, a third booster SARS-CoV-2 vaccine shot has been administered in many Chinese cities. This study aimed to explore the association of vitamins A, D, and E with dynamic changes in neutralizing antibody titers (wild-type and omicron) after the third booster shot in a prospective cohort and provided evidence and clues for nutrition education during the COVID-19 pandemic.

## 2. Materials and methods

### 2.1. Study population

**Figure 1:**
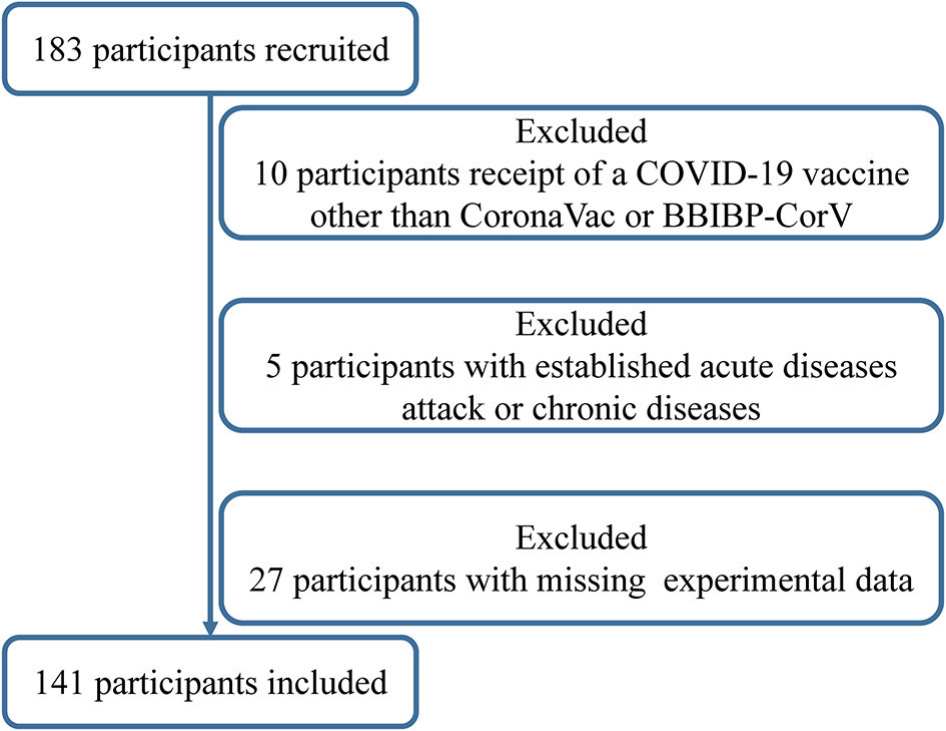
Flowchart of the participants' enrollment process.

We conducted a prospective, open-label study (chictr.org.cn identifier: ChiCTR2200059259) at Zhuhai People's Hospital in Zhuhai, China, to explore the relationship of vitamins A, D, and E with dynamic changes of neutralizing antibody titers within 98 days after the third homologous boosting shot of inactivated SARS-CoV-2 vaccine (CoronaVac; Sinovac or BBIBP-CorV; Sinopharm) in healthy adults aged 18–59 years from December 2021 to April 2022. The subjects entered the study according to the inclusion criteria: no past or current SARS-CoV-2 infection had received two doses of inactivated whole-virion vaccines more than 6 months, and women were not pregnant or puerperal. A total of 183 subjects met the inclusion criteria and were invited to participate in the study, and 42 subjects were excluded according to established criteria: receipt of COVID-19 vaccine other than CoronaVac or BBIBP-CorV; allergy to any ingredient of vaccines; acute diseases attack or chronic diseases with/without acute exacerbation (including uncontrolled hypertension, diabetes complications, malignant tumor, renal diseases, and known autoimmune disease); the appearance of 10 symptoms of COVID-19 such as fever, cough, runny nose, and sore throat within 7 days before the third boost with the vaccine; using immunosuppressive medications and vitamin supplements for 15 days before and after the vaccine; a shot of other vaccines 14 days before the third vaccine or other vaccines planned within 28 days; having participated in other clinical studies; missing experimental data at any time point; and any condition that could interfere with the primary objectives. Written informed consent had been obtained from all participants before the enrolment (Figure 1). The study's informed consent and protocol were reviewed and approved by the Ethics Committee of Zhuhai People's Hospital.

### 2.2. Vaccination procedure and blood collection

**Figure 2:**
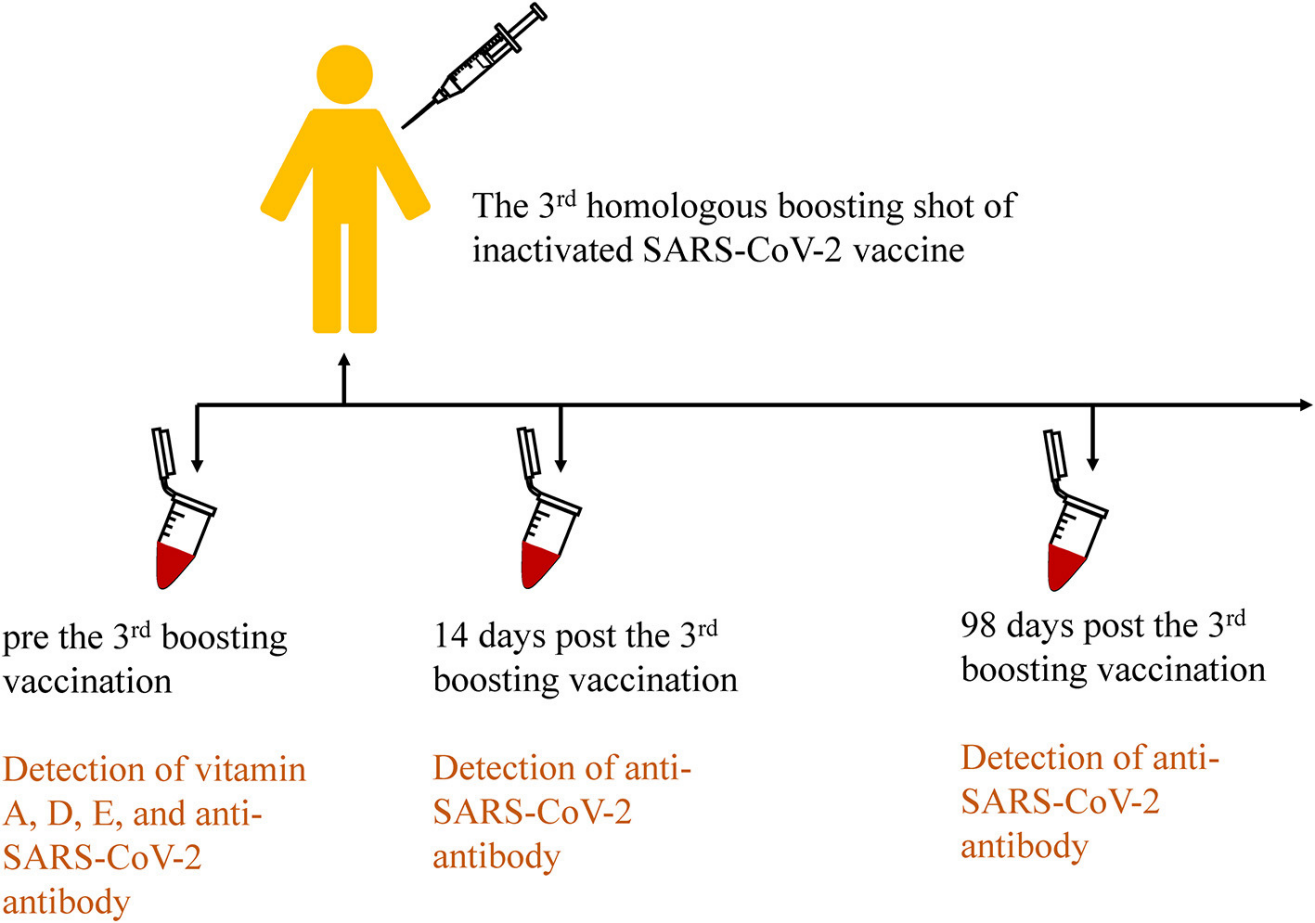
Procedures of the third homologous boosting shot of inactivated SARS-CoV-2 vaccination and sample collection in the cohort study.

All subjects received the third booster shot inactivated SARS-CoV-2 vaccines (CoronaVac or BBIBP-CorV, according to the previous vaccination program) more than 6 months after the second shot. All subjects underwent three blood draws that were handled according to the standards of practice before the third inoculation, 14 days, and 98 days after inoculation, respectively. The procedure is shown in Figure 2. Next, plasma was separated from blood cells immediately, and then aliquots (20 μL) were pipetted onto the imprinted circles of the dried plasma spots (DPS) card for vitamin analysis. The spots were dried for 2 h in the dark and then stored at −20°C with desiccant in resealable aluminum foil bags until analysis. The remaining plasma was stored at −80°C for further analysis.

### 2.3. Detection of vitamins A, D, and E

Plasma vitamin concentrations were measured using high-performance liquid chromatography-tandem mass spectroscopy (LC-MS/MS, Nexera UHPLC LC-30A, and SCIEX Triple Quad™ 6500+), online coupled with fully automated dried blood spot extraction (CAMAG DBS-MS 500). DPS cards were moved by a robotic arm from the racks toward the different workstations, including an optical recognition system (locating the position of the spots on the cards), an IS module (spraying of an IS solution onto the cards before extraction), and an extraction module containing a 4 mm clamp head for clamping of a DPS card. A total of 10 μL internal standard composed of retinol-d5, 25(OH)D_3_-13C5, and α-tocopherol-d6 in acetonitrile was sprayed onto the cards. Subsequently, 70% methanol aqueous solution as the extraction solvent is horizontally guided through the clamped area of the DPS, and the resulting extract is then sent into a sample loop. The autosampler is equipped with a wash station to avoid cross-contamination between subsequent samples.

For LC-MS/MS analysis, the optimized mass transition ion pairs (m/z) for quantitation were 269.2/93 for retinol, 274.2/93 for retinol-d5, 383.3/257 for 25(OH)D_3_, 388.5/262.3 for 25(OH)D_3_-13C5, 431.3/165 for α-tocopherol, and 437.3/171 for α-tocopherol-d6. The chromatographic separation was achieved on a Phenomenex Kinetex PFP (4.6 mm × 100 mm, 2.6 μm) column with a flow rate of 0.6 mL/min, using gradient elution with acetonitrile and 0.1% formic acid 0.05% heptafluorobutyric acid in water as the mobile phase. For quality check, each batch contained 48 samples with four quality control samples inserted, and intra- and inter-batch coefficients of all vitamins were below 15%. All participants had plasma vitamins above the quantitation limit (4.0 ng/L for retinol, 1.0 ng/L for 25(OH)D_3_, and 10.0 μg/L α-tocopherol).

### 2.4. Detection of the anti-SARS-CoV-2 receptor-binding domain neutralizing antibody

At the same time, we evaluated the anti-RBD responses in fasting blood samples at three-time points above by the serum surrogate virus neutralization test (sVNT) to assess the dynamic changes of the neutralizing antibody. Circulating NAb against SARS-CoV-2 was detected which blocked the interaction between the RBD of the viral spike glycoprotein with the angiotensin-converting enzyme 2 cell surface receptor in the experiment. Recombinant S-RBD from the wild type (Wuhan-Hu-1) and omicron (B.1.1.529) strains were used in this study. All experimental operations and the use of the SARS-CoV-2 sVNT kit (GenScript) were performed according to the manufacturer's instructions. Tests were performed on Varioskan Lux (ThermoFisher).

For all assays, the limit of quantitation (LOQ) was 9.38 U/mL, and levels < LOQ were substituted with LOQ/Sqr(2).

### 2.5. Statistical analysis

Demographic characteristics of the study are summarized as mean ± standard deviation (SD) or median and interquartile range (IQR) for continuous variables, as no. (%) for categorical variables. Neutralizing antibody titers are presented as geometric mean titers (GMTs) with 95% confidence intervals (CIs). The seropositivity rate was defined as the serum anti-SARS-CoV-2 antibody concentrations exceeding 4 × LOQ. The Kolmogorov–Smirnov test was used to assess the normality of variables and those not normally distributed were log-transformed. The Mann–Whitney *U*-test was used to compare the difference in anti-SARS-CoV-2 neutralizing antibody growth and decay between subgroups. The Spearman correlation test was used to assess the association of antibody growth and decay with the target vitamin concentrations. We performed multivariate linear regression (anti-wide-type antibody as the dependent variable) and logistic regression (anti-omicron antibody seropositivity or not as the dependent variable) analyses to assess the relationships between antibody levels and the concentrations of plasma retinol, 25(OH)D_3_, and α-tocopherol. Statistical analyses were performed using R software (version 4.1.3), with the two-sided significant level at 0.05.

## 3. Results

### 3.1. Study participants characteristics

The distribution of the participants' characteristics is presented in Table 1. After excluding participants with missing experimental data, 141 were included in the study. The mean age of patients was 30.16 years, and the mean body mass index (BMI) was 22.01 kg/m^2^; 54.61% of patients had regular physical activity, and 71.63% were injected with the BBIBP-CorV vaccine. Over 90% of participants were of Han ethnicity, did not smoke or drink, and had a college education or higher.

**Table 1:** Characteristics of the study participants by sex group.

**Variable**	**Total (*N =* 141)**	**Male (*N =* 49)**	**Female (*N =* 92)**
Age, years, mean ± SD	30.16 ± 7.84	29.35 ± 7.24	30.59 ± 8.14
Race, Han, no. (%)	136 (96.45)	48 (34.04)	88 (62.41)
BMI, kg/m^2^, mean ± SD	22.01 ± 4.15	23.73 ± 3.12	21.10 ± 4.34
**Smoking no. (%)**			
Never	133 (94.33)	42 (29.79)	91 (64.54)
Former	1 (0.71)	1 (0.71)	0
Current	7 (4.96)	6 (4.26)	1 (0.71)
**Drinking no. (%)**			
Never	136 (96.45)	46 (32.62)	90 (63.83)
Former	1 (0.71)	1 (0.71)	0
Current	4 (2.84)	2 (1.42)	2 (1.42)
**Education levels no. (%)**			
Middle school	3 (2.13)	0	3 (2.13)
High school	4 (2.84)	1 (0.71)	3 (2.13)
College or above	134 (95.04)	48 (34.04)	86 (61.00)
Physical activity, yes, No. (%)	77 (54.61)	34 (24.11)	43 (30.50)
First-to-second dose interval, days, median (IQR)	31.00 (22.00–41.00)	29.00 (18.00–41.00)	31.00 (23.00, 40.25)
Second-to-third dose interval, days, median (IQR)	252.00 (200.00–293.00)	257.00 (207.00–296.00)	245.50 (197.00–291.25)
**Manufacturer of vaccine no. (%)**			
BBIBP-CorV	101 (71.63)	37 (26.24)	64 (45.39)
CoronaVac	40 (28.37)	12 (8.51)	28 (19.86)

SD, standard deviation; IQR, interquartile range.

### 3.2. Distribution of anti-wild-type virus and anti-omicron variant antibody titers at the baseline and 14 and 98 days after the third booster dose

We detected anti-wild-type virus and anti-omicron variant antibodies at the baseline and on days 14 and 98 after the third vaccination (Table 2). We found that the third vaccination induced a significantly higher degree of humoral immunogenicity for either the wild-type or omicron variant (days 14 and 98 after the third vaccination vs. baseline, *P* < 0.001, respectively). The third vaccination induced not only a significantly high humoral immunogenicity of the wild-type virus but also of the omicron variant. The seropositivity rates were 98.58%, 100.00%, and 100% for the anti-wild-type virus antibodies and 11.34%, 50.35%, and 35.46% for the seropositivity rates of anti-omicron variant antibodies at the baseline and on days 14 and 98, respectively (*P* < 0.001, compared with the baseline).

**Table 2:** Distribution of antibody titers against wild-type and Omicron at three-points time.

	**Baseline**	**14 days after 3rd vaccination**	**98 days after 3rd vaccination**
**Prototype, U/mL**			
GMT (95% CI)	142.43 (127.74–159.17)	831.34 (742.48–925.19)	436.27 (391.51–482.99)
Seropositivity rate	98.58%	100.00%	100.00%
textitP^*a*^ value		< 0.001	< 0.001
**Omicron, U/mL**			
GMT (95% CI)	11.93 (10.18–14.01)	31.03 (25.03–38.47)	20.19 (16.78–24.29)
Seropositivity rate	11.34%	50.35%	35.46%
*P^*a*^* value		< 0.001	< 0.001

GMT, geometric mean titer; IQR, interquartile range; LOQ, limit of quantitation.

The seropositivity rate was defined as at least a 4-fold higher than the limit of quantitation of the assays.

a The P-value of the t-test.

### 3.3. Associations of retinol, 25(OH)D_3_, and α-tocopherol with anti-wild-type virus and the anti-omicron variant neutralizing antibodies

**Figure 3:**
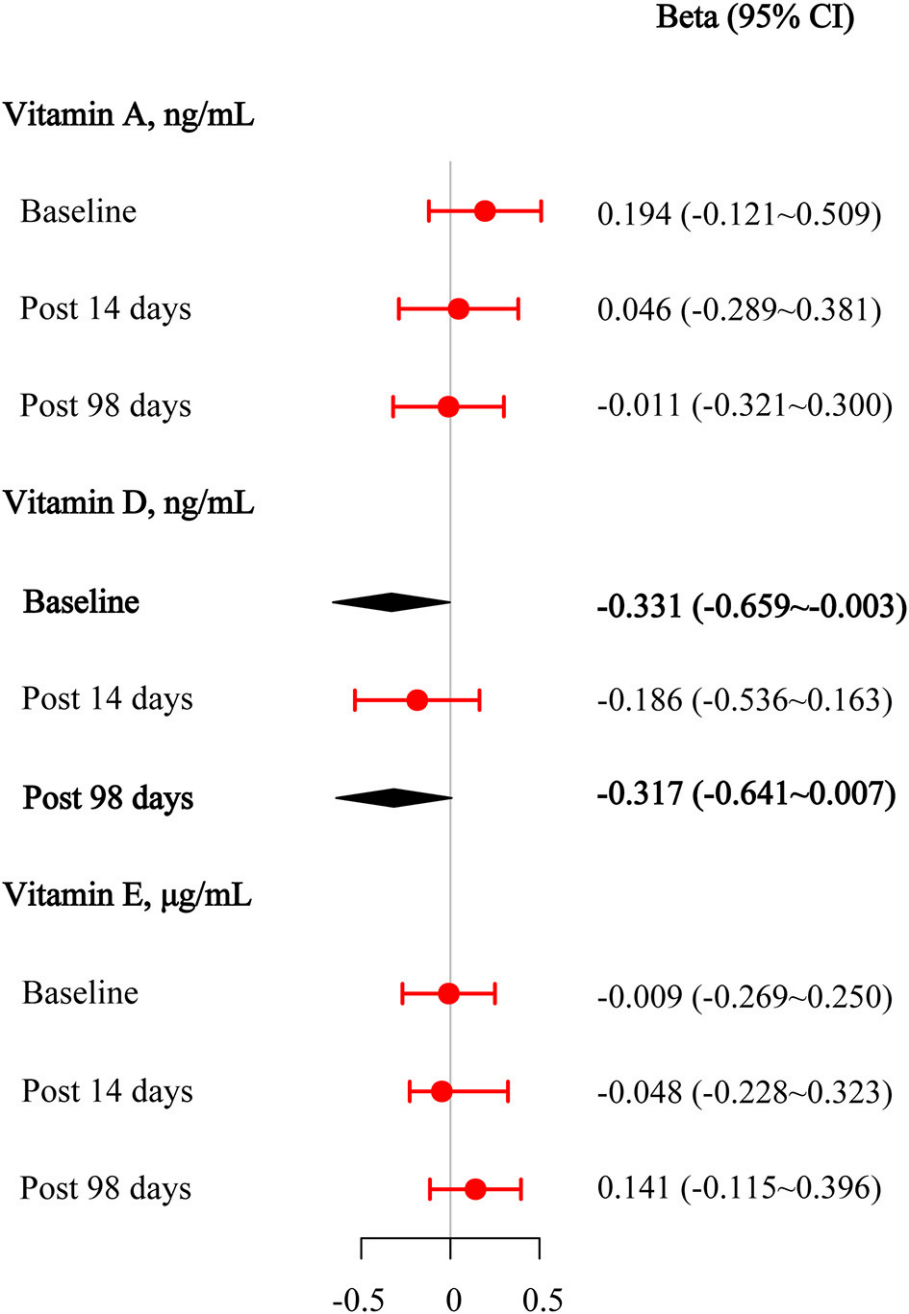
Multivariable linear regression results among vitamins A, D, and E for anti-wild-type virus-neutralizing antibody titers at the baseline, 14 days after the third vaccination, and 98 days after the third vaccination.

We performed multivariate regression analyses to assess the effect of the target vitamin levels at the baseline on anti-wild-type virus-neutralizing antibodies before and after the third booster dose. The results are presented in Figure 3. After adjusting for age, sex, BMI, physical activity status, the intervals between the first and second vaccination and between the second and third vaccination, and the manufacturer of the vaccine, we found that 25(OH)D_3_ was significantly negatively associated with baseline anti-wild-type virus antibody concentrations (beta [95% CI] = −0.331 [−0.659–−0.003]). After adjusting for covariates, there was a potentially negative association between 25(OH)D_3_ and anti-wild-type virus antibody concentrations on day 98 after the third vaccination (beta [95% CI] = −0.317 [−0.641–0.007]). No statistically significant associations were observed between retinol and anti-wild-type virus antibody concentrations after adjusting for covariates (all *P* > 0.050).

**Figure 4:**
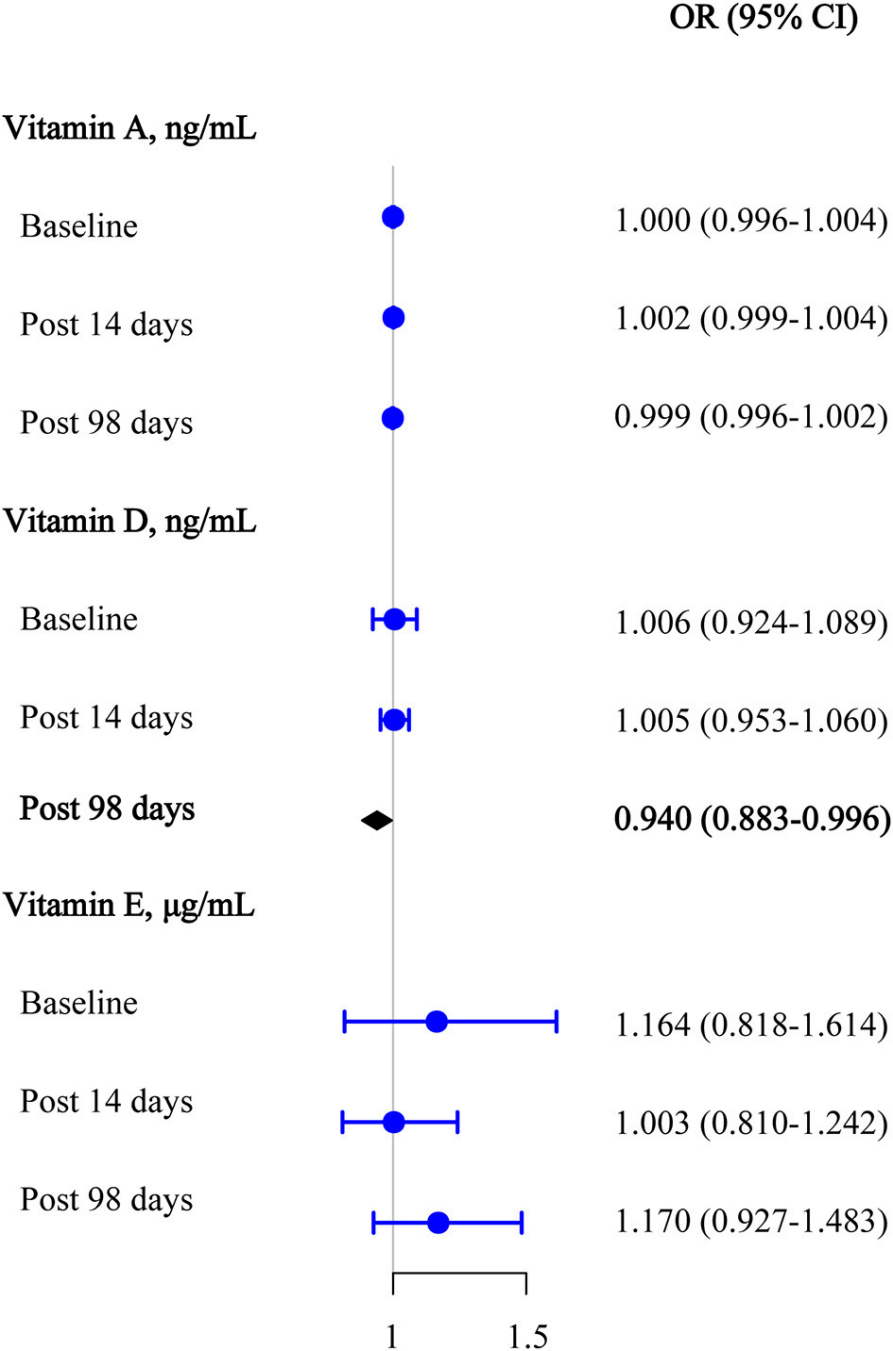
Multivariable logistic regression results among vitamins A, D, and E for anti-omicron variant neutralizing antibody seropositive at the baseline, 14 days after the third vaccination, and 98 days after the third vaccination.

Logistic regression analyses were used to assess the effect of target vitamin levels at the baseline on the seropositivity of anti-omicron variant neutralizing antibodies before and after the third booster dose (Figure 4). After adjusting for covariates, we also found that 25(OH)D_3_ was significantly negatively associated with the seropositivity of the anti-omicron variant antibody upon day 98 after the third vaccination (OR [95% CI] = 0.940 [0.883–0.996]). Similarly, no statistically significant associations were observed between the retinol and α-tocopherol and the seropositivity of the anti-omicron variant antibodies after adjusting for covariates (all, *P* >0.050).

Subgroup analyses showed an association between 25(OH)D_3_ and the baseline anti-wild-type virus antibody concentrations, which seemed to be more pronounced among subjects with the female, with the intervals between the first and second vaccination ≤ 30 days (Supplementary Figure 1A). The association between 25(OH)D_3_ and the anti-wild-type virus antibody concentrations at day 98 after the third vaccination seemed to be more pronounced among subjects with the intervals between the first and second vaccination being >30 days (Supplementary Figure 1B). We also found the association of 25(OH)D_3_ with the seropositivity of anti-omicron variant antibody at day 98 which after the third vaccination seemed to be more pronounced among subjects with male, CoronaVac-vaccinated, with the intervals between the first and second vaccination being >30 days and the intervals between 2nd and 3rd vaccination being >240 days (Supplementary Figure 1D).

To further explore the relationship between target vitamins and changes in anti-wild-type virus-neutralizing antibodies on days 14 and 98 after the third vaccination, we assessed the correlations between target vitamins, fold increases in antibody titers (comparison between the baseline and 14 days after the third vaccination), and fold decreases in antibody titers (comparison between 14 days after the third vaccination and 98 days after the third vaccination). However, we observed no correlation between the target vitamins and fold increase/decrease in antibody titers (all *P* > 0.050; Supplementary Table 2).

## 4. Discussion

In this study, we used a prospective, open-label design to assess the relationships between vitamins A, D, and E nutritional status and neutralizing antibodies (anti-wild-type and anti-omicron) before and after the third inactivated SARS-CoV-2 vaccination. The third vaccination induced a significantly high degree of humoral immunogenicity for both the wild-type virus and the omicron variant. We found that plasma vitamin D levels were significantly negatively associated with the baseline anti-wild-type virus antibody levels, potentially negatively associated with anti-wild-type virus antibody concentrations on day 98 after the third vaccination, and significantly negatively associated with the seropositivity of the anti-omicron variant antibody on day 98 after the third vaccination. The inverse associations between plasma vitamin D and anti-wild-type virus antibody levels and seropositivity of the anti-omicron variant antibodies persisted in the subgroup analyses. We observed no significant association between vitamin A/E and anti-wild-type virus antibody levels or seropositivity of the anti-omicron variant antibodies.

The inactivated SARS-CoV-2 vaccine is widely used to prevent infection and severe COVID-19, and neutralizing antibody levels are vital predictors of vaccine efficiency (22). Similar to the results of Ai et al., the third homologous booster vaccination enhanced participants' immune responses against SARS-CoV-2 (23). However, the neutralizing capacity against the omicron variant was significantly lower than that against the wild-type virus, confirming the results of another study (24).

Numerous previous studies have found that low vitamin D levels were associated with significantly higher antibody titers after antiviral vaccination in people with a relatively high vitamin D nutritional status, which is consistent with our study. Linder et al. found significantly higher mean geometric rubella antibody titers in winter-inoculated children than in summer-inoculated children; vitamin D was stimulated by ultraviolet radiation (25). A study on HPV vaccines in college-aged men suggested that antibody titers for all HPV strains were significantly higher in individuals with lower vitamin D levels than in those with higher vitamin D levels (11). A cross-sectional study found that low serum vitamin D levels were associated with higher antibody titers against partial influenza virus vaccines in children (26). Another cross-sectional analysis of the National Health and Nutrition Examination Survey (NHANES) highlighted a negative association between serum 25(OH)D levels and measles antibody titers (27). Many studies have demonstrated the expression of the vitamin D receptor (VDR) in almost all immune cells (28). Therefore, vitamin D has potent direct effects on active B lymphocytes (i.e., VDR expression is upregulated) (29). Vitamin D inhibits immunoglobulin production through various mechanisms, including the inhibition of cytokine-mediated B-cell activation by acting on T-helper cells, suppressing the differentiation of mature B cells into plasma cells and class-switched memory B-cells, and inducing apoptosis of both activated B- and plasma cells (30).

However, the effect of vitamin D on the immunogenicity of vaccines is complex, and the association between vitamin D and antibody response to COVID-19 vaccines is inconsistent in the current research. Several studies have reported no clear correlation between vitamin D levels and antibody responses to anti-SARS-CoV-2 mRNA vaccination (31, 32). A sub-study nested within the CORONAVIT randomized controlled trial also reported that vitamin D supplementation did not influence the protective efficacy or immunogenicity of SARS-CoV-2 mRNA or adenovirus vaccinations in old adults (33). Moreover, a few studies reported results contrary to ours, suggesting that adequate levels of vitamin D may improve the antibody response to SARS-CoV-2 mRNA vaccines (34, 35). A differential immune response has been observed for different SARS-CoV-2 vaccine types (36); therefore, vitamin D potentially has different effects on different vaccine types. Rubella, HPV, influenza, and measles vaccines, as well as the anti-SARS-CoV-2 vaccine used in our study, are all inactivated antiviral vaccines. Vitamin D deficiency can cause dysregulation of the immune response (37), and correcting this deficiency can effectively improve the immune response. Positive associations between vitamin D and antibody response to the SARS-CoV-2 vaccine were obtained by comparing the adequate status of vitamin D and insufficient/deficiency status (34, 35). However, positive associations were not observed in another study of older adults (33), which might have been caused by different population backgrounds; the former included middle-aged people with better immune status and stronger immune responses. In our study, the subjects were mainly middle-aged people with better immune status and stronger immune response and had a relatively high vitamin D nutritional status; <30% of patients had vitamin D insufficiency [i.e., a 25(OH)D level of 20–30 ng/mL], and none had vitamin D deficiency [i.e., a 25(OH)D level <20 ng/mL] (38). Additionally, the genetic and ethnic backgrounds of different populations should be considered (39, 40).

No significant associations were observed between vitamins A and E and the antibody response of both wild-type and omicron variant disease following inactive anti-SARS-CoV-2 vaccination in our study; this result was consistent with the results of previous studies. Gardner et al. found that vitamins A and E were not associated with antibody responses to influenza vaccines in healthy elderly individuals (41). An observational prospective cohort study reported that vitamins A and E levels were not related to the odds of seroprotection or seroconversion to the influenza vaccine in older adults (19). A prospective randomized controlled clinical trial suggested that vitamins A and E supplementation did not affect the IgG response to tetanus toxoid in healthy children (17). However, a clinical trial suggested that weekly maternal vitamin A supplementation during pregnancy and postpartum could enhance prenatal H1N1-vaccine responses in mothers with low vitamin A status (42). In our study, only approximately 2% of subjects had vitamin A deficiency [retinol level <200 ng/mL as vitamin A deficiency (43)], approximately 95% of subjects had vitamin E deficiency [α-tocopherol level <5 μg/mL as vitamin E deficiency (44)], and none had vitamin A or E supplementation. Therefore, the association of vitamins A and E with the antibody response to inactive anti-SARS-CoV-2 vaccination needs to be verified in individuals with different vitamin A and E statuses.

Our study has several advantages. Our study is a prospective cohort study to estimate the anti-wild-type virus and anti-omicron variant antibody response in the medium-to-long-term following the third inactive anti-SARS-CoV-2 vaccination. It is also, to the best of our knowledge, the first to explore the associations between vitamins A, D, and E with anti-wild-type virus/anti-omicron variant antibody response to inactive anti-SARS-CoV-2 vaccination. This study reveals the relationship between vitamin D and antibody response, which provides new clues for precise nutrition during the COVID-19 pandemic, especially for the present omicron pandemic. However, this study had several limitations. First, most subjects had a relatively high vitamin D and vitamin A nutritional status, and most subjects had a relatively poor vitamin E nutritional status; therefore, we were unable to assess the effects of different vitamin A, D, and E nutritional statuses on the antibody response. Second, the study did not include people who used vitamin supplements to further explore the effects of vitamin supplementation on the antibody response. Third, our subjects were mainly aged 24–42 years, which meant that they had a better immune status and stronger immune response; therefore, younger and older people were ignored. Finally, different vaccine types and populations with different genetic backgrounds should be considered because of the complex effects of vitamins on immune responses. In the future, the relationship between vitamins A, D, and E and antibody response to inactive anti-SARS-CoV-2 vaccination should be comprehensively described in different populations.

## 5. Conclusion

Our study suggested that the third homologous boosting vaccination enhanced subjects' immunity response against SARS-CoV-2, and low vitamin D levels were associated with significantly higher antibody titers for the anti-wild-type virus and higher antibody seropositivity for the anti-omicron variant after the third inactive anti-SARS-CoV-2 vaccination in people with adequate levels of vitamin D, better immune status, and stronger immune response; further studies comprising large cohorts of patients with different nutritional status are warranted to verify our results.

## Data availability statement

The original contributions presented in the study are included in the article/Supplementary material, further inquiries can be directed to the corresponding authors.

## Ethics statement

The studies involving human participants were reviewed and approved by the Ethics Committee of Zhuhai People's Hospital. The patients/participants provided their written informed consent to participate in this study.

## Author contributions

YD, TS, LH, JL, and PL conceived the study, performed manuscript revision, and took accountability for all aspects of the work. YD, LH, and PL designed the methodology and did the software analysis. TS and JL were in charge of supervision and administration. All authors performed the data interpretation, drafted and revised the manuscript, and read and agreed to the published version of the manuscript.
