# Supplementary material for: Association of fat-soluble vitamins (A, D, and E) status with humoral immune response to COVID-19 inactivated vaccination
Source: Front Nutr. 2023 May 16;10:1167920. doi: 10.3389/fnut.2023.1167920 (PMC10227435; doi:10.3389/fnut.2023.1167920)
Supplement: Supplementary file 1 [file Data_Sheet_1.docx]

Supplementary Material

Association of fat-soluble vitamins (A, D, and E) status with humoral immune response to COVID-19 inactivated vaccination

**Yao Deng ^1†^, Liting Huang ^1†^, Peixin Liu ^2†^, Xuyang Geng ^1^, Zefang Lin ^1^, Zhixiong Zheng ^1^, Meixiao Zhan ^1^, Zhiren Zhang ^1^, Junwei Liu ^1,3*^, Taoping Sun ^1*^**

*** Correspondence:**

Taoping Sun: Zhuhai Precision Medical Center, Zhuhai People’s Hospital, Zhuhai Hospital Affiliated with Jinan University, 79 Kangning Rd, Zhuhai 519000, China. Tel/Fax: +86 0756 2158603. Email: d201578100@alumni.hust.edu.cn.

Junwei Liu: Zhuhai Precision Medical Center, Zhuhai People’s Hospital, Zhuhai Hospital Affiliated with Jinan University, 79 Kangning Rd, Zhuhai 519000, China. Tel/Fax: +86 0756-2158603. Email: ljw700616@126.com.

# Supplementary Figures and Tables

## Supplementary Figure

**
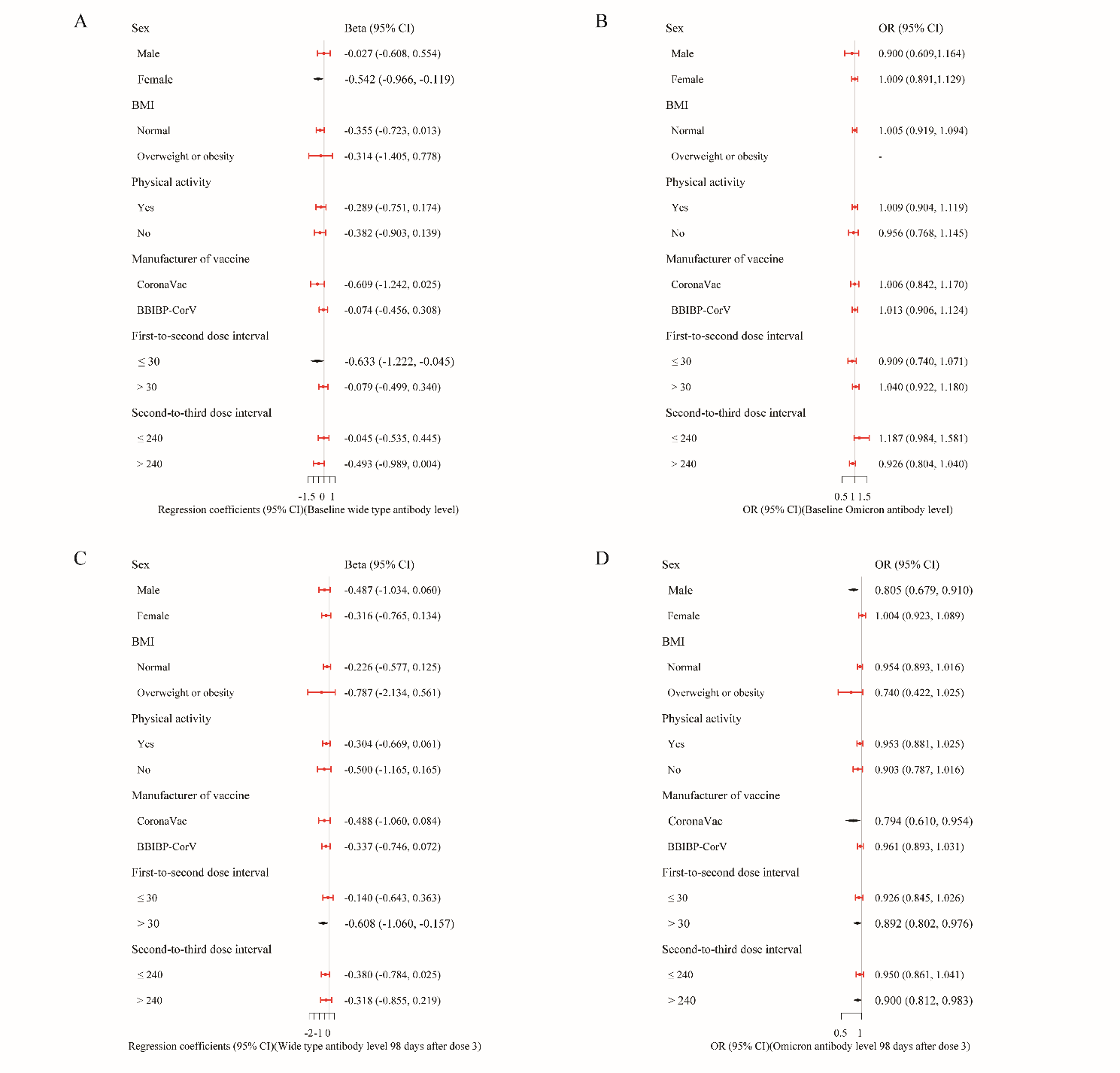
**

**Supplementary Figure 1.** Stratified analyses of anti-wide-type virus neutralizing antibody titers and plasma vitamin D at baseline (A) and 98 days after 3rd vaccination (C), anti-Omicron variant neutralizing antibody seropositive and plasma vitamin D at baseline (B) and 98 days after 3rd vaccination (D).

## Supplementary Table

| **Supplementary Table 1 The distribution of vitamins A, D, and E at three-time points before and after the third inactivated SARS-CoV-2 vaccination.** | | | | | | | | |
| --- | --- | --- | --- | --- | --- | --- | --- | --- |
|  | **Vitamin A, ng/mL** | | **Vitamin D, ng/mL** | | | **Vitamin E, μg/mL** | |  |
|  | **median (IQR)** | | **median (IQR)** | | | **median (IQR)** | |  |
| Baseline | 394.60 (286.40-498.60) | | 37.36 (29.20-44.89) | | | 3.54 (2.85-4.82) | |  |
| Post 14 days | 409.00 (306.00-513.90) | | 39.36 (32.29-48.02) | | | 3.25 (2.33-4.35) | |  |
| Post 98 days | 366.40 (282.20-471.60) | | 41.73 (34.98-49.78) | | | 2.59 (1.87-3.19) | |  |
| Abbreviations: IQR, interquartile range. | | | | | | | |  |
|  | | | | | | | |  |
| **Supplementary Table 2 Associations of the variables and the fold-changes of the antibody titers.** | | | | | | | | |
| **Variable** | | **Spearman's correlation coefficient (antibody growth)** | | ***P* value** | **Spearman's correlation coefficient (antibody decay)** | | ***P* value** | |
| Age | | -0.064 | | 0.454 | 0.047 | | 0.584 | |
| BMI, kg/m^2^ | | 0.025 | | 0.771 | 0.030 | | 0.726 | |
| Intervals between 1^st^ and 2^nd^ vaccination | | 0.110 | | 0.197 | -0.043 | | 0.613 | |
| Intervals between 2^nd^ and 3^rd^ vaccination | | -0.005 | | 0.951 | 0.117 | | 0.167 | |
| Sex | | - | | 0.873 | - | | 0.130 | |
| Physical activity | | - | | 0.718 | - | | 0.241 | |
| Manufacturer of vaccine | | - | | 0.178 | - | | 0.076 | |
| Vitamin A, ng/mL | | -0.033 | | 0.698 | 0.083 | | 0.329 | |
| Vitamin D, ng/mL | | 0.042 | | 0.619 | -0.016 | | 0.854 | |
| Vitamin E, μg/mL | | 0.024 | | 0.783 | 0.075 | | 0.375 | |
